# The impact of using electromyographic biofeedback on pelvic floor rehabilitation in men with post-prostatectomy urinary incontinence: a meta-analysis

**DOI:** 10.1016/j.clinsp.2025.100687

**Published:** 2025-05-13

**Authors:** Camila Chaves dos Santos Novais, Adélia Regina Oliveira da Rosa Santana, Alisson Rodrigo Moura da Paz, Aline Tenório Lins Carnaúba, Kelly Cristina Lira de Andrade, Pedro de Lemos Menezes

**Affiliations:** a Northeast Biotechnology Network (RENORBIO), Universidade Federal de Alagoas (UFAL), Maceió, Alagoas, Brazil; b Faculdade Estácio de Alagoas, Maceió, AL, Brazil; c Universidade Estadual de Ciências da Saúde de Alagoas (UNCISAL), Maceió, AL, Brazil

**Keywords:** Prostatectomy, Urinary incontinence, Physiotherapy, Biofeedback

## Abstract

**Objective:**

To identify the impact of using electromyographic biofeedback on pelvic floor rehabilitation in men with post-prostatectomy urinary incontinence.

**Materials and methods:**

Searches in the databases were carried out: Pubmed/Medline, Latin American and Caribbean Literature in Health Sciences, Physiotherapy Evidence Database, Cochrane Library, Embase, Scopus, Web of Science, and in gray literature. The study included randomized clinical trials that treated men with post-prostatectomy urinary incontinence using electromyographic biofeedback in pelvic rehabilitation compared to other resources or no treatment. Studies including incontinent men with sphincter implants, pharmacological treatments, or studies with incomplete data were excluded. Assessment of risk of bias using the Critical Appraisal Tool developed by Joanna Briggs Institute for randomized clinical trials and the GRADE approach to assess the certainty of evidence of meta-analysis results were used.

**Results:**

16 articles were included, of which, 7 studies were subjected to two meta-analyses to assess the relative risk of men becoming continent and the reduction of urine loss through pad. Participants who received treatment showed a 1.78 times greater risk of achieving continence compared to those who did not receive treatment (RR = 1.78; 95% CI 1.29‒2.45; I^2^ = 77%). The limitations of the present study are the lack of high-quality evidence, in which existing trials suffered from a lack of standardized outcome measures.

**Conclusion:**

Electromyographic biofeedback in pelvic rehabilitation seems to contribute to the faster achievement of continence in prostatectomized men compared to those who did not undergo any intervention. Additionally, helps to reduce pad weight.

## Highlights

• Urinary incontinence is observed as the main post-prostatectomy complication.
• Pelvic floor rehabilitation is the first-line treatment for urinary incontinence.
• Electromyography biofeedback instructs the patient on the correct activation muscle.

## Introduction

Prostate Cancer (PCa) ranks as the fourth most prevalent cancer worldwide. It is estimated that 1.4 million new cases are diagnosed annually, accounting for 15.2% of all cancers in men. This rate corresponds to an estimated risk of 31.50 per 100,000 individuals. The highest incidences of PCa are found in Northern Europe, Western Europe, the Caribbean, and Oceania (North and East).^1^

In the Brazilian context, PCa is the second most common type of cancer among men in all regions, surpassed only by non-melanoma skin cancer. Considering both genders, it ranks fourth in absolute terms, with an estimated 71,730 new cases for the 2023‒2025 period. It represents an estimated risk of 67.86 new cases per 100,000 men.^1^

Early diagnosis of prostate cancer has shown a significant reduction in mortality by approximately 30%. Surgical procedures such as radical prostatectomy, radiotherapy, and chemotherapy provide a chance of cure. Radical Prostatectomy (RP) stands out with a cure rate of up to 94%. However, this intervention is associated with complications such as Urinary Incontinence (UI) and sexual dysfunction that significantly impact the patient's psychological, physical, social, economic, and sexual spheres.^2^

UI is a significant concern after RP. Internationally defined as any involuntary loss of urine by the International Continence Society (ICS), post-RP prevalence ranges from 1% to 87%, depending on the assessment period, surgical technique, preoperative condition of the patient, and assessment instrument.^3^ Post-RP incontinence is attributed to deficiency or injury caused by surgery to the urethral sphincter, bladder dysfunction such as detrusor overactivity and/or decreased bladder compliance, and injury to the innervation responsible for muscular control of urethral closure and/or bladder function.^4^, ^5^, ^6^, ^7^

Complications of post-RP UI have profound effects on social and professional life. Shame and embarrassment lead to social isolation while professionals’ repercussions include job loss, with some patients requiring government assistance during their recovery period. Psychologically, patients may experience depression, embarrassment, and low self-esteem, while financial impacts involve additional expenses for personal hygiene products such as diapers and treatments.^2^^,^^8^

Pelvic rehabilitation, led by a physiotherapist, has emerged as a conservative approach for treating UI, as it offers the advantage of not causing side effects and promotes faster recovery through Pelvic Floor Muscle Training (PFMT), which is responsible for urethral closure.^6^ First-line treatment for UI, PFMT, when performed regularly, improves motor function due to the recruitment of phasic muscle fibers that stimulate the contraction reflex, and increases the tone of tonic fibers, providing recovery from urinary dysfunction.^9^, ^10^, ^11^

Electromyography Biofeedback (EMG-BFB) stands out as a crucial tool in muscle training, aiming to capture muscular electrical activity and instruct the patient on correct muscle activation through visual or auditory feedback. This method improves coordination and muscle strength.^12^

The objective of this systematic review is to identify the impact of using electromyographic biofeedback on pelvic floor rehabilitation in men with post-prostatectomy urinary incontinence.

## Methods

This systematic review was conducted in accordance with the Preferred Reporting Items for Systematic Reviews and Meta-Analyses (PRISMA/2020).^13^

### Eligibility criteria

To establish the eligibility criteria, the acronym PICOS was used: P = Population (prostatectomized men with urinary incontinence); I = Intervention (electromyographic biofeedback); C = Comparison (any other physiotherapeutic resource or no treatment); O = Outcomes (urinary continence); S = Study design (randomized clinical studies).

### Inclusion criteria

The study included randomized clinical trials that treated men with post-prostatectomy urinary incontinence using electromyographic biofeedback in pelvic rehabilitation compared to other resources or no treatment. Outcomes were continence, based on the mean number of used pads and mean pad weight in grams; muscular strength measured by the degree of strength or electromyographic value and quality of life. No language or publication date restrictions were used.

### Exclusion criteria

Studies that included incontinent men with sphincter implants, comparison of pharmacological treatments, and studies with incomplete data were excluded. Articles not found in full were excluded after no response was received from the authors following contact attempts by the reviewers.

### Information sources and search strategies

Searches were carried out in the following databases: Pubmed/Medline, Latin American and Caribbean Literature in Health Sciences (LILACS), Physiotherapy Evidence Database (PEDro), Cochrane Library, Embase, Scopus, Web of Science, and in gray literature such as Google Scholar and ProQuest. A manual search of the references of the included articles was also performed, and an expert on the subject was consulted to recommend any relevant articles. The searches were carried out on July 3, 2023.

The search strategy was developed by crossing keywords using Health Sciences Descriptors (DeCS) and Medical Subject Headings (MeSH) descriptors and their synonyms combined with the Boolean operators AND and OR (Appendix 1). References were managed and duplicates were removed using Endnote® software (EndNote® X7 Thomson Reuters, Philadelphia, PA).^14^

### Study selection

The selection of studies was carried out by two independent reviewers (CN and AP) and the Rayyan® website^15^ was used to store and select the articles in a blinded manner. Disagreements were resolved through discussion between the reviewers. In cases the two reviewers could not reach an agreement, a third reviewer (AC) was consulted.

To ensure correct calibration between reviewers, before starting phase 1 reading, a pre-selection of articles was independently carried out based on a partial search of the literature, and the Kappa agreement coefficient was calculated. The first reading stage only started after obtaining agreement values > 0.7 between the two reviewers.^16^

### Extracting data and data items

Data regarding the results and primary characteristics of the selected studies were extracted, including first author, year of publication, country of study, sample size, application of electromyographic biofeedback in the treatment group, the resource or therapy used (or the absence of treatment) control group, and follow-up time. Follow-up time was expressed in weeks or months after surgery. All information was independently collected by two authors (CN and AP), aiming to ensure data accuracy and integrity. Any discrepancies between the two authors were resolved by discussion.

The primary variables analyzed in the studies were the number of continent men, the mean number of pads used, and the mean pad weight in grams, using the Pad Test. Furthermore, muscle strength was assessed through numerical mean, using the Strength Scale or electromyographic values. For outcomes where data were expressed as frequency, values for the number of events and total sample were collected for each group. When data were reported using continuous data, mean and standard deviation values for each group and the total sample were extracted from the text.

The secondary analyzed variable was quality of life or the impact of incontinence on quality of life, expressed in numerical values obtained from scores in the questionnaires used in the studies.

### Risk assessment of bias

Methodological quality analysis was performed using the JBI Critical Appraisal Tools developed by Joanna Briggs Institute for randomized clinical trials^17^ (Appendix 3). The assessment domains were judged as “yes”, “no”, “uncertain”, or “not applicable” for the answers to each question. The percentage of domains judged as “Yes” was used as an instrument for the categorical assessment of each study. “Yes” answers up to 49% of the total, the risk of bias was considered high. When 50% to 69% were “yes”, or moderate, and when more than 70%, low risk of bias.^18^ The JBI tool criteria were examined separately by two reviewers (CN) and (AP). The third investigator (AC) was consulted in cases of discrepancies.

#### Effect measures

For variables presented through binary data, the relative risk was used as a measure of effect, while for variables treated with continuous data, the difference between the means was calculated. In both effect measures, a 95% Confidence Interval (95% CI) was considered for the global estimate.

#### Synthesis method

A meta-analysis was conducted using a random effect model, weighted by the inverse variance method for continuous variables, and the Mantel-Haenszel method for binary variables. This analysis was performed using the RStudio statistical software, version 1.2.1335 (RStudio Inc., Boston, USA).^19^ To calculate the variance, expressed by Tau^2^, the DerSimonian-Laird estimator was used, heterogeneity was calculated by the Inconsistency Index (I^2^), and the significance level was set at 5%. A minimum of three articles was established with the necessary data that met the eligibility criteria for quantitative synthesis for each outcome.

#### Assessment of reporting bias

An assessment of publication bias using a funnel plot and Egger's test was planned. However, due to the limited number of studies available (n < 10), it was not possible to carry out this approach. To minimize the possibility of publication bias, a broad search was carried out in several databases, including the gray literature, in addition to the LILACS database, which covers publications in languages other than English.

### Certainty of evidence assessment

The level of certainty of evidence was assessed using the Grading of Recommendations, Assessment, Development, and Evaluation (GRADE) tool.^20^ This tool classified the generated evidence into four levels of certainty: very low, low, moderate, and high, considering the following assessment domains: study limitations (risk of bias), inconsistency, indirectness, imprecision, and publication bias.

## Results

### Study selection

**Figure 1:**
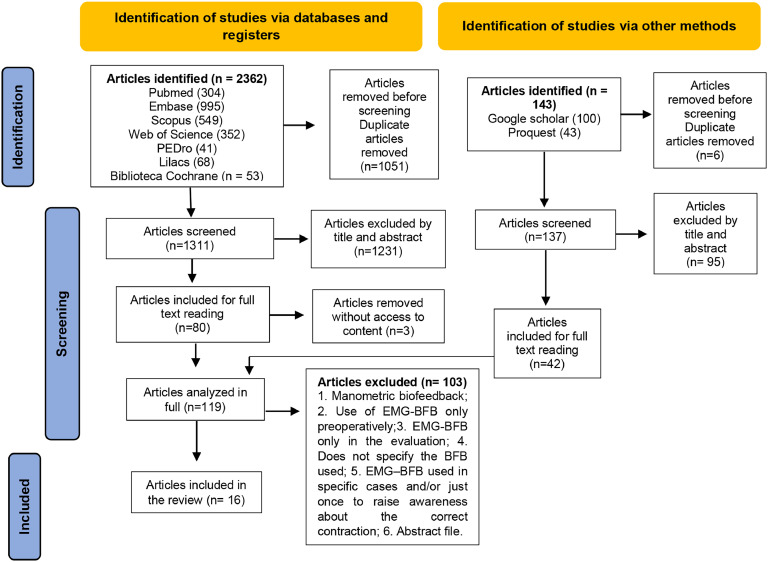
PRISMA flowchart. Caption: flowchart elaborated for the study selection process using PRISMA. Source: prepared by the authors.

During the final database search, a total of 2505 articles were identified, of which 1448 remained after eliminating duplicates. After reviewing the titles and abstracts in the initial phase, 80 articles were selected for full reading. Among these, three were excluded as they were not available in full, despite three attempts to contact the authors via email sent once a week, for three weeks. Additionally, 42 articles were included from the gray literature and expert consultations in the next phase, totaling 119 articles for analysis. Of these, 103 were excluded as detailed in Appendix 2, resulting in 16 articles selected for qualitative synthesis (Fig. 1). No additional articles were found during the manual search of references.

### Characteristics of the studies

Of the 16 included studies, three were carried out in Italy,^21^, ^22^, ^23^ two in the USA^24^^,^^25^ and two in Poland,^26^^,^^27^ one in Canada,^28^ Egypt,^29^ Iran,^30^, Brazil,^31^ China,^32^ Spain,^33^ Germany,^34^ the Netherlands^35^ and Belgium.^36^ English was the predominant language of studies, used in 15 studies.

The size of the samples ranged from 30 to 180 participants. In total, there were 1332 men with UI after RP, with a mean age of 64.4±4.8 years, and follow-up time ranging from 3 weeks to 12 months.

Information regarding the characteristics of the included studies is shown in Table 1.

**Table 1:** Description of articles included in the review.

Authors, Year/Country	Assessment measures/instruments	Treatment protocol/ n° of sample	Findings
Ahmed; Muhammad; Amansour, 2012, Egypt	24-hour pad test; quality of life with questionnaire (IIQ-7). Reassessed at 6-, 12- and 24-weeks.	**CG (26) =** Oral instructions and informative leaflet; **ESG (26) =** 2 × week, for 12-weeks. **BFB+ES G (28) =** 2 × week for 12 weeks (**n** = 80).	The mean pad weight decreased significantly (p < 0.05) in the BFB+ES group compared to the others at 6-, 12-, 24-weeks. There was a significant increase (p < 0.05) in the continence rate in BFB + ES G at 6- and 12-weeks. Significant improvement in the impact of UI recorded IIQ-7 in the BFB+ES group compared to the other groups.
Allameh et al., 2021 / Iran	24-hour pad test. Reassessed at 1, 3 and 6 months.	**Group 1 (18): EMG-BFB** 2 × week, for 2-weeks (pre-op). Post-operative NF probe. **Group 2 (19):** Preoperative NF probe, **EMG-BFB** 2 × week, for 2-weeks (post-operative). **Group 3 (18):** Preoperative and postoperative NF probe (**n** = 55).	The mean number of pads per 24-hours showed no statistical differences between the groups at 1-month, but after 3- and 6-months the number was higher in G3.
An et al., 2021, China	1-hour pad test; Recording the number of incontinence episodes in daily life; ICIQ-SF for subjective assessment of UI; Oxford rating scale for determining PF strength recovery. Reassessed at 4- and 8-weeks.	**Group A (14)**: Kegel training; **Group B (14)**: Kegel training combined with **EMG-BFB; Group C (14)**: **EMG-BFB** combined with Pilates training. Every day for 8-weeks (**n =** 42)	At 8-weeks, GA, GB and GC showed an improvement of 34%, 61% and 67% in the 1-hour pad test, respectively. Reduction of 32%, 52% and 58% in the number of UI episodes, respectively; reduction of 29%, 50% and 64% on the ICIQ-SF scale; gain in muscle strength of 33%, 50% and 50% on the Oxford scale, respectively.
Floratos et al., 2002, Netherlands	1-hour pad test and a questionnaire to determine the n° of pads per day. Reassessed at 1-, 2-, 3- and 6-months.	**Group A (28): EMG-BFB** 3 × week, for 5-weeks. **Group B (14)**: Oral and written guidance through a leaflet with instructions for exercises at home. (**n =** 42).	There was no statistically significant difference in the patients' level of incontinence between the groups. The continence rate after 6-months was 91%.
Franke et al., 2000, USA	Voiding diary and 48-hour pad test. Reassessed at 6-, 12- and 24-weeks after surgery.	**TG:** 5 sessions with **EMG-BFB** (6-, 7-, 9-, 11- and 16-weeks), home exercises. **CG**: no treatment and no exercise guidance **(n = 30).**	The training effect provided a mean increase of 34% in pelvic muscle work at the end of the 16-weeks. 54% and 70% were continent at 3-months in the treatment and control groups, respectively. 87% of patients were continent within 6-months. There was no statistically significant difference in the number of UI episodes and pad weight between the groups.
Geraerts et al., 2013, Belgium	24-hour and 1-hour pad test; Visual Analogue Scale (VAS), International Prostate Symptom Score (IPSS) and quality of life (King's Health Questionnaire-KHQ. Assessed before RP and reassessed at 1-, 3-, 6- and 12-months after RP.	**SG (91): PFMT** with BFB 3 weeks before surgery; BFB continued after surgery. **CG (89): PFMT** with BFB after catheter removal. Post-operatively. All patients attended the hospital 1 × week to perform 1 individual guided exercise session with digital BFB or EMG control (**n** = 180)	The mean time to continence was 30- and 31-days, and the mean amount of incontinence on the first day was 108g and 124g for SG and CG, respectively. And the “impact of incontinence” (KHQ) was better for SG at 3-months and 6-months after surgery.
Goode et al., 2011, USA	Voiding diary for 7-days. Reassessed after 8-weeks of treatment.	**Behavioral therapy (70)**: PF muscle training and bladder control strategies. **Behavioral therapy plus (70)**: in-office, **EMG-BFB** and daily home pelvic floor EE. **Late treatment (68):** which served as a control group. All groups were followed for 4 visits approximately 2-week interval (**n** = 208)	Mean incontinence episodes decreased from 28 to 13 per week after behavioral therapy, and from 26 to 12 after behavior plus therapy. Both reductions were significantly higher than the reduction from 25 to 21 observed within control group (p = 0.001 for both treatment groups). There was no significant difference in incontinence reduction between treatment groups (p = 0.69). Gains lasted up to 12-months in the active treatment groups: 50% reduction in the behavioral group and 59% reduction in the behavior plus group (p = 0.32).
Mariotti et al., 2015, Italy	24-hour pad test and number of pads used. Initial assessment, 14-days after catheter removal in (Group 1) and 12-months after surgery in (Group 2), reassessed at 2 and 4-weeks and at 2, 3, 4, 5 and 6-months.	**G1 (60) early treatment:** BFB + FES 14-days after tube removal 2 × week, for 6-weeks. **G2 (60) late treatment:** 12-months after surgery, same procedure as G1 (**n** = 120)	The mean leak weight was significantly reduced (p < 0.002) in Group 1 compared to Group 2 from visit 1 to visit 7. A significant difference (p < 0.05) was observed between the two groups for the percentage of continent patients only at 2-weeks (Group 1 = 20%; Group 2 = 0%), and 4 weeks (Group 1 = 66.7%; Group 2 = 46.7%).
Mariotti et al., 2009, Italy	24-hour pad test and the incontinence section of the International Continence Society questionnaire. Reassessed at 2- and 4-weeks and at 2-, 3-, 4-, 5- and 6-months.	**G1 (30): EMG-BFB** + FES 2 × week, for 6 weeks. **G2 (30)**: Oral and written instructions on exercises (**n** = 60)	The mean leak weight was significantly reduced (p = 0.05) in Group 1 compared to Group 2, starting at 4-weeks through 6-months of follow-up.
Moore et al., 2008, Canada	24-hour pad test. The IPSS, IIQ-7 questionnaires, cost of treatment and perception of urine loss as a problem. Assessed at 4-weeks after RP, reassessed at 8-, 12-, 16-, 28- and 52-weeks.	**CG (99):** Oral instructions and leaflet with exercises at home. **IG (106): PFMT + BFB** once a week up to 24-weeks. Oral instructions and leaflet with exercises at home (**n** = 166).	No significant differences were found between groups in grams of urine loss or proportion of continent participants. At the IPSS, there was no statistically significant difference between the groups, however the mean difference from baseline for all individuals to 12-months was statistically significant (p < 0.001). IG reported a slight improvement in QoL, but it was not statistically significant.
Rajkowska-Labon et al., 2014, Poland	1-hour and 24-hour urinary pad tests; Voiding diary and incontinence questionnaire; Tension measurements of the PF muscles were applied using EMG (surface electromyography). The duration of therapy depended on the level of incontinence and continued for no more than 12-months.	**IG: IGA (23) PFMT** + **EMG-BFB** 1 × week. and **PFMT** + exercises with principles of stabilization of the vertebral segment 1 × week. and guidelines for exercising at home. **IGB (26) PFMT** + exercises with principles of stabilization of the vertebral segment 2 × week. and guidelines for performing exercises at home. **IGI (32):** control group without treatment (**n** = 81)	There was a statistically significant difference between the IA versus IB subgroups (p = 0.007) with a higher percentage of continent patients in IB 24/26 (92%). Superior continence results were obtained in IG × IGI (33 × 4) p = 0.0001. EMG-BFB only changed the muscular response time between continent and incontinent individuals (p = 0.03).
Ribeiro et al., 2010, Brazil	24-hour pad test; incontinence symptoms measured by ICSI; lower urinary tract symptoms measured by ICST; the impact of incontinence on quality of life measured by the IIQ-7. Reassessed at 1-, 3-, 6-, 12-months.	**TG (26): PFMT** + **EMG-BFB** 1 × week, for a maximum of 12-weeks. **CG (28)**: Did not receive formal education on PF exercises. Oral instructions by urologist to contract PF (**n** = 54)	The duration of incontinence was shorter in the treatment group. At the 12^th^ postoperative month, 25 (96.15%) patients in the treatment group and 21 (75.0%) in the control group were continent (p = 0.028). Significant change in both groups in UI symptoms (ICSI) and lower urinary tract symptoms (ICST), IIQ7 quality of life and muscle strength (p = 0.001) for all parameters.
Soto Gonzalez et al., 2020, Spain	1-hour and 24-hour pad test; ICIQ-SF; Voiding diary. Reassessed after 1-, 2-, 3- and 6-months.	**TG (25):** Physiotherapy with electrotherapy and biofeedback, 3 × week, for 3-months, **CG (22)**: did not receive any specific treatment. Both groups received a printed leaflet for performing pelvic floor exercises at home (**n** = 47)	The 1-hour pad test showed statistically significant differences between the groups at 3-months (p = 0.001) and at 6-months (p = 0.001), improved for the TG. 64% of patients in the TG recovered continence compared to 9% in the CG. The 24-hour pad test showed a significant difference between the groups after 3-months (p = 0.003) and 6-months (p = 0.001), better for the TG. ICIQ-SF showed significant differences between the groups at 2-months (p = 0.014), 3-months (p = 0.001) and 6-months (p = 0.0001), improved for the TG.
Szczygielska et al., 2022, Poland	1-hour pad test. Reassessed after completing treatment (10-weeks).	**Groups: A (20) PFMT** and **B** (**20) PFMT** + **EMG-BFB** (random division, RP time: 2– 6 weeks) and **Group C (20)** = **PFMT** (RP time > 6-weeks). All performed 1x week, for 10-weeks (**n =** 60)	UI improved in all groups: A, p = 0.0000; B, p = 0.0000; and C, p = 0.0001. After PFMT completion, complete control of urination was achieved by 60% of patients in group A, 85% in group B and 45% in group C.
Tienforti et al., 2012, Italy	Number of incontinence episodes and number of pads; the questionnaires: UCLA-PCI, ICIQ-OAB and IPSS-QoL. Reassessed at 1-, 3- and 6-months.	**CG (16):** oral and written instructions on pelvic floor exercises. **GI (16):** one preoperative session with **EMG-BFB** and monthly postoperative visits at 1-, 3- and 6-months. And a structured exercise program to be carried out at home (**n** = 32).	In the intervention group, continence was achieved by 6, 8 and 10 patients at 1-, 3- and 6- months of follow-ups, respectively, versus 0 (p = 0.02), 1 patient (p = 0.01) and 1 patient (p = 0.002) in the control group at each follow-up, respectively. Analysis of the UCLA-PCI and ICIQ-OAB scores, the number of incontinence episodes and the number of pads per week showed significant differences for patients in the intervention group at 3- and 6-months.
Zellner M., 2011, Germany	International Prostate Symptom Score (IPSS); Pad test; Record of the number of pads used; Pelvic floor contraction force through a rectal probe. Reassessed at 3- to 4-weeks.	**Group S (25):** Individual and group physiotherapy; **Group G (25)**: Individual and group physiotherapy + EE+ **EMG-BFB; Group F (25)**: group G + body vibration protocol. 2 × week, for 3 to 4-weeks (**n** = 75).	There was improved quality of life in all 3 treatment groups. Group S improved by 1.8, Group F by 1.7 and Group G by 1.4 points. They were statistically significant for all 3 treatment groups (p < 0.0001). There was a statistically significant reduction in urine volume in the vibration group GFpre = 40.7g, GFpost = 10.9g; p < 0.0001.

IIQ-7, Incontinence Impact Questionnaire, Short Form; CG, Control Group; ESG, Electrical stimulation group; BFB+ES G, Biofeedback and Electrical Stimulation Group; UI, Urinary Incontinence; G1, Group 1; EMG-BFB, Electromyographic Biofeedback; p/w, per week; NF, Non-Functional; n, number; ICIQ-SF, International Consultation on Incontinence Questionnaire-Short Form; PF, Pelvic Floor; TG, Treatment Group; FES, Functional Electrical Stimulation; PFMT, Pelvic Floor Muscle Training; EMG, Electromyography; EE, Electrostimulation; IG, Intervention Group; ICS, International Continence Society; ICSI, Incontinence Symptoms of the International Continence Society male Short Form questionnaire; ICST, Total score of the International Continence Society male Short Form questionnaire; RP, Radical prostatectomy; UCLA-PCI, University of California, Los Angeles Prostate Cancer Index; ICIQ-OAB, International Consultation on Incontinence Questionnaire - Overactive Blade; IPSS-QoL, International Prostate Symptom Score Quality of Life; QoL, Quality of life.

Source: Prepared by the authors.

### Risk of bias assessment

According to the risk of bias analysis, nine studies^21^^,^^22^^,^^24^^,^^26^^,^^27^^,^^31^^,^^33^, ^34^, ^35^ were classified as having a moderate risk of bias. This was due to the non-concealment of participant allocation, and the lack of evaluator blinding and the individual responsible for conducting the procedures. A total of seven studies^23^^,^^25^^,^^28^^,^^29^^,^^30^^,^^32^^,^^36^ were classified as having a low risk of bias. Out of the 16 studies, 13 were categorized as not applicable for participant blinding, as the treatment approach involved executing exercise, which made blinding unfeasible.

**Figure 2:**
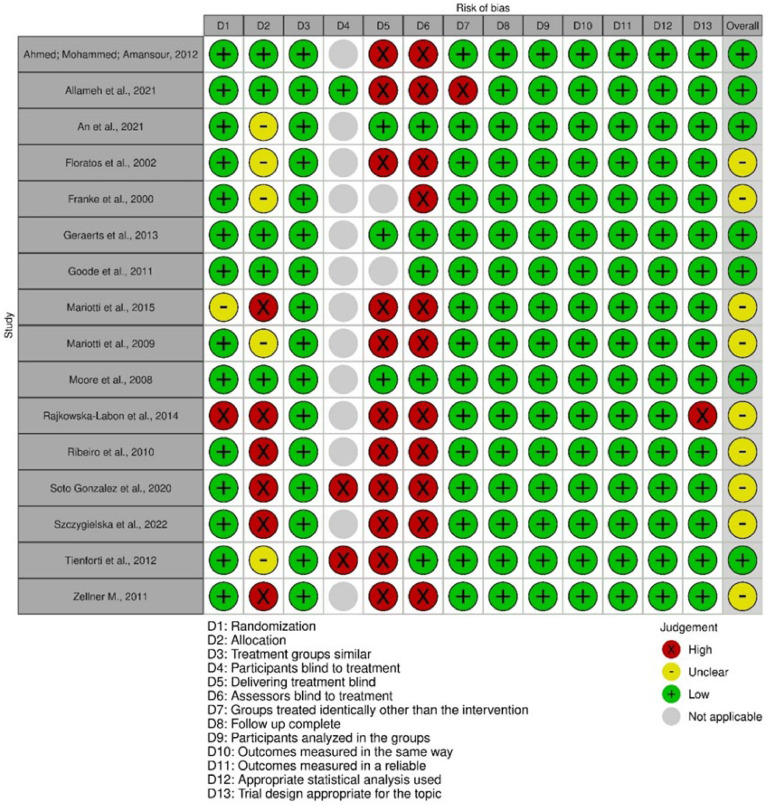
Analysis of the methodological quality of the article. Caption: methodological quality scheme: review of the authors' judgments on each methodological quality item for each included study. Source: prepared by the authors.

Figure 2 shows the risk of bias assessment of the articles included in this review.

### Individual study results

Number of continent men, frequency and intensity of UI, quality of life and muscle strength

In relation to the number of continent men, frequency, and intensity of UI, the application of the 1-hour and/or 24-hour pad test and voiding diary recording is observed. To assess quality of life and the impact of UI on quality of life, the following questionnaires were generally used: ICIQ-SF, International Consultation on Incontinence Questionnaire ‒ Short Form; IIQ-7, Incontinence Impact Questionnaire, Short Form; ICSI, Incontinence symptoms of the International Continence Society male Short Form questionnaire; KHQ, King's Health Questionnaire; UCLA-PCI, University of California, Los Angeles Prostate Cancer Index; ICIQ-OAB, International Consultation on Incontinence Questionnaire-Overactive Bladde; IPSS-QoL, International Prostate Symptom Score Quality of Life. Only four studies assessed the pelvic floor muscle strength using the Oxford scale^31^ and measurement of muscle tension by EMG.^24^^,^^26^^,^^34^

### Intervention

The studies developed in two groups were: EMG-BFB × oral and written instructions;^28^^,^^31^^,^^35^ EMG-BFB × no treatment or guidance;^24^ Preoperative and postoperative EMG-BFB × postoperative EMG-BFB;^36^ EMG-BFB immediate postoperative period for 6-weeks × late postoperative period after 12-months;^21^ EMG-BFB and electrical stimulation × oral and written instructions;^22^ EMG-BFB and electrical stimulation × no treatment;^33^ preoperative with EMG-BFB and in monthly postoperative visits × oral and written instructions.^23^

The studies were conducted in three groups: Electrostimulation × BFB and electrostimulation × oral instructions and leaflet;^29^ EMG-BFB for 2-weeks (pre-operative) and non-functional probe (post-operative) × non-functional probe pre-operatively and EMG-BFB for two weeks post-operative × non-functional probe pre-operative and post-operative;^30^ Pelvic muscle exercise × pelvic muscle exercise and EMG-BFB × EMG-BFB and pilates;^32^ PFMT × EMG-BFB and electrical stimulation × no treatment;^25^ EMG-BFB and PFMT and exercises with stabilization of the vertebral segment × PFMT and exercises with stabilization of the vertebral segment × no treatment;^26^ PFMT × PFMT and EMG-BFB (RP time: 2– 6-weeks) x PFMT (RP time > 6-weeks);^27^ Individual and group physiotherapy × Individual and group physiotherapy and electrostimulation and EMG-BFB × Individual and group physiotherapy and electrostimulation and EMG-BFB and body vibration.^34^

There was an improvement in UI signs in which the number of continent men was higher in the EMG-BFB intervention groups compared to other interventions or controls without intervention.^23^^,^^29^^,^^31^^,^^33^ An improvement in quality of life was also observed in the questionnaires applied.^24^^,^^30^^,^^32^, ^33^, ^34^^,^^36^ However, studies^24^^,^^25^^,^^34^^,^^35^ did not show statistically significant differences between intervention and control groups in reducing incontinence. EMG-BFB contributed mainly to improving muscle response time with increased muscle work, and greater muscle strength.^24^^,^^26^^,^^32^ Detailed data from each study are described in Table 1.

### Result of the synthesis

Data from seven studies were subjected to two meta-analyses to assess the relative risk of men becoming continent and the reduction of urine loss, measuring pad weight, and using rehabilitation with EMG-BFB or without treatment (only oral and written guidelines).

**Figure 3:**
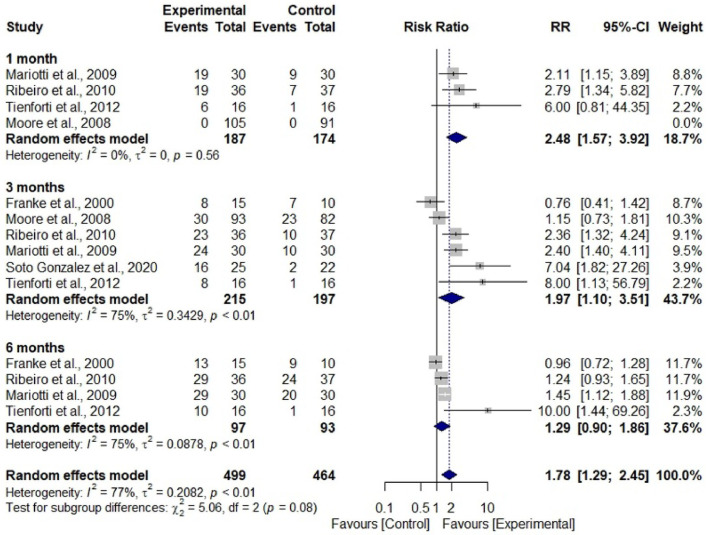
Forest Plot to assess the number of men who achieved continence during the rehabilitation period with electromyographic biofeedback associated with pelvic muscle training compared to the group without treatment. Caption: number of continent men assessed in periods between experimental and control groups. Source: prepared by the authors.

**Figure 4:**
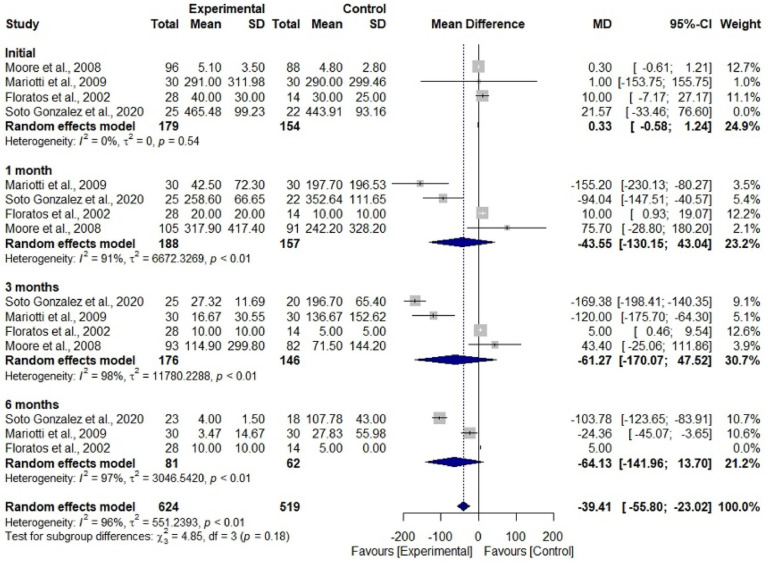
Forest Plot to assess pad weight in grams per period of rehabilitation with electromyographic biofeedback associated with pelvic muscle training compared to the group without treatment. Caption: mean and standard deviation of the pad weight in grams assessed in periods between the experimental and control groups. Source: prepared by the authors.

Participants who had undergone treatment showed a 1.78 times greater risk of achieving continence compared to those who had not undergone any treatment (RR = 1.78; 95% CI 1.29–2.45; I² = 77%). This finding suggests that participants who are not treated have approximately 44% less risk of achieving continence compared to those who do not (Fig. 3). Regarding the weight of the pad, there was no difference between the considered subgroups; however, there was a significant difference in overall estimate, with a mean difference of -39.41 (95% CI -55.80–-23.02; I^2^ = 96%), in which the experimental group presented lower mean values compared to the control group (Fig. 4).

### Reporting bias

Due to the number of articles included (7 studies out of 16), it was not feasible to assess publication bias using the funnel plot or the Egger test.

### Certainty of evidence

Certainty of evidence was classified as low for the meta-analysis number of continent men and also low for pad weight. The decrease in certainty of evidence was due to the diversity of treatment protocols and follow-up time, with a serious risk of bias and serious inconsistency, as described in Table 2.

**Table 2:** Detailed data from each study.

Certainty assessment							Number of patients		Effect		Certainty
Number of continent men											
N° of studies	Study design	Risk of bias	Inconsistency	Indirect	Imprecision	Other Considerations	EMG-BFB	No treatment	Relative (95% CI)	Absolute (95% CI)	
6	Randomized clinical trials	Serious^a^	Serious^b^	Not serious	Not serious	None	234/499 (46.9%)	124/464 (26.7%)	**RR 1.78** (1.29 to 2.45)	**21 more per 100** (from 8 more to 39 more)	⨁⨁◯◯ Low
Pad Weight											
4	Randomized clinical trials	Serious^a^	Serious^c^	Not serious	Not serious	None	624	519	‒	MD **39.41 grams lower** (55.8 lower to 23.02 lower)	⨁⨁◯◯ Low

CI, Confidence Interval; MD, Mean Difference; RR, Risk Ratio.

a Presence of studies with moderate risk of bias.

b Presence of heterogeneity in the analysis (I-square = 77%).

c Presence of heterogeneity in the analysis (I-square = 96%). Source: Prepared by the authors.

## Discussion

This systematic review assessed the evidence on the impact of EMG-BFB in the rehabilitation of prostatectomized men with UI. Seven studies were included for meta-analysis, of which five were at moderate risk of bias^22^^,^^24^^,^^31^^,^^33^^,^^35^ for presenting 50% to 69% of the respondents responding ‘yes’ to each question in the Critical Appraisal Tool developed by the Joanna Briggs Institute for randomized clinical trials; and two had a low risk of bias^23^^,^^28^ for presenting 70% or more ‘yes’ answers to each question.

Most of the included studies had high risk in the following domains: no concealment of participant allocation, blinding of evaluators, and blinding of those responsible for conducting the procedures. Such assessment was due to the exercise treatment in the intervention group versus the control group without intervention, which makes blinding these domains difficult to apply.

This meta-analysis could only be performed with two urinary continence variables: number of continent men in six studies^22^, ^23^, ^24^, ^28^, ^31^, ^33^ and absorbent weight in four studies^22^^,^^28^^,^^33^^,^^35^ due to the great methodological variability between the studies.

A relationship was observed between a greater number of continent men in the treatment group compared to the control group at all follow-up times 1-month, 3-months, and 6-months.

While the analysis of the pad weight showed imprecision in all subgroups of times, in the final result, precision was observed in favor of the treatment group. The results of the subgroups may be related to the low number of included studies; variation in the application of the pad test, in which three studies applied the 24-hour test, one 1-hour test; different treatment protocols; and follow-up time which ranged from 12 to 24 sessions.

Current definitions of continence range from no leaks, and no pads, to loss of a few drops of urine, or 1 safety pad per day, while severe incontinence is defined as frequent and severe urinary loss. Post-prostatectomy UI is generally multifactorial and may vary depending on patient characteristics, preoperative continence status, sphincter competence, pre-and postoperative detrusor muscle function and surgical conditions.^37^

The pad test is the most objective way to quantify urine loss over a given period of time. The application time may range from short duration (20 minutes), to 1-hour-long, 24-hour, 48-hour and 72-hour duration. In these tests, the 24-hour test proved to be reliable, reproducible and superior to the 1-hour test and more practical than the 48-hour and 72-hour tests as they are difficult to adhere. Additionally, the level of performed activities and the amount of liquid consumption throughout the test may also affect adherence.^38^

EMG is described as the extracellular recording of bioelectrical activity generated by muscle fibers. Despite capturing the electrical activity promoted by the recruitment of motor units and not muscle strength, there is a good correlation between the number of activated motor units and muscle strength. Thus, strong muscles provide better support for the demands of increased intra-abdominal pressure in daily activities without causing urine leakage.^39^

EMG-BFB provides the patient, through feedback signals (visual and/or sound), with information about the execution of the training and directs the correct muscle activation more effectively, promoting self-regulation of exercise strength, power, and resistance. Pressure biofeedback is influenced by the abdominal pressure exerted during exercise as it cannot objectively isolate only the capture of pressure exerted on the pelvic muscle probe.

The contribution of EMG-BFB with PFMT seems to be associated with a faster achievement of continence observed from the data in Figure 3, in the periods of 3-months and 6-months, and the reduction in the pad weight observed in the joint mean.

In the meta-analysis carried out by Kannan (2018)^40^ who analyzed various approaches, one of the analyses was the effectiveness of PFMT in combination with BFB compared to the control without treatment. The study identified a greater number of men who were continent in the intervention group immediately after intervention in comparison to the control group (63/194 vs. 38/180 in the control group); however, the effect was not statistically significant (RR = 1.70 [95% CI 0.95 to 3.04]; p = 0.07). Additionally, a greater number of continent men in follow-up were found in the intervention group than in the control group (131/178 vs. 104/167 in the control group); however, the effect was not statistically significant (RR = 1.17 [95% CI 0.93 to 1.48]; p = 0.18). They also found no statistically significant differences between the groups by weight in the 24-hour pad test immediately after the intervention (-94.54 [95% CI ÿ433.38 to 244.30]; p = 0.58; n = 250) or at follow-up (-9.29 [95% CI -44.47 to 25.89]; p = 0.60; n = 221.

The current review made a specific analysis of the use of EMG-BFB in PFMT versus a control group without intervention, which differs from the review carried out by Kannan et al., 2018,^40^ which analyzed several procedures such as PFMT versus no intervention, PFMT with electrostimulation versus no intervention and sham electrostimulation, PFMT with BFB and electrostimulation versus sham electrostimulation, and also PFMT with BFB versus a control group no intervention, the latter with findings similar to the present review. However, the fact that they did not specify which type of BFB was analyzed in the meta-analysis makes the analysis unspecific since pressure BFB and EMG differ in the way pelvic floor muscle activity is captured and recorded.

Hsu et al., 2016^41^ performed meta-analysis to evaluate the subjective improvement of UI, identifying statistically significant differences in the medium and long-term effects (mean effect size = 0.226 and 0.278; 95% CI: 0.44 to 0.02 and 0.47 to 0.08; p = 0.034 and 0.005, respectively, when comparing the PFMT + BFB group with PFMT alone. Evidence on immediate effects was lacking (mean effect size = 0.13; 95% CI: 0.29 to 0.03; p = 0.108). An objective analysis of UI improvement through the pad test showed significant immediate, intermediate, and long-term effects of PFMT + BFB compared to PFMT alone (all p < 0.05).

This meta-analysis analyzed two interventions that observed evidence in the treatment of UI assessed both by self-report and by pad weight, which differentiates the analysis groups compared to the present meta-analysis, as it compared a group with PFMT instead of a control group without treatment, it also does not specify the type of BFB used in the studies analyzed.

The systematic review conducted by MacDonald et al.^42^ observed that, in five studies involving 348 men, significantly more men in the PFMT + BFB group achieved continence or were without ongoing leaks than in the group without training or usual care at 1–2 months after RP, at 57% vs. 37% (relative benefit increase 1.54; 95% CI 1.01–2.34; four trials) in pooled analysis. All five studies provided data 3 to 4 months after RP; pooled analysis showed that the relative increase in benefit was not significant (1.19; 0.82–1.72), with 87% and 69% of the treatment group and control group achieving continence, respectively. PFMT with BFB was compared to written/oral instruction in three trials with 281 men. No study reported a significant difference between the groups for any outcome at any of the assessed times.

The present review considered the oral/written guidelines as a group without treatment since both the BFB treatment and non-treatment groups received guidance on pelvic anatomy, changes in habits and exercises for the pelvic floor muscles at home, diverging from the analysis carried out by MacDonald^42^ who carried out an analysis with a group without treatment or usual care and another analysis with a group with oral/written instructions. The first analysis obtained similar findings to this study and the second did not. Another important factor to be highlighted is the failure to specify the BFB type.

The limitations of the present study are the lack of high-quality evidence on the use of EMG-BFB for UI after prostate surgery. Existing trials suffered from a lack of standardized outcome measures, varying in the application of pad testing (1-hour and 24-hour tests) and duration of self-reporting of the voiding diary to record the number of pads, quality of life analysis demonstrated with the low number of articles included in the meta-analysis, and the low certainty of evidence variables analyzed.

## Conclusion

EMG-BFB in pelvic rehabilitation suggests a contribution to the faster achievement of continence in prostatectomized men with a higher risk than in men who did not undergo any intervention. The treatment also shows reduced pad weight.

Post-prostatectomy UI is a distressing problem and although there are no studies of high methodological quality, conservative interventions such as EMG-BFB are frequently used in pelvic rehabilitation. However, robust randomized clinical trials are needed, with standardized outcome measures and improved methodological quality to assess both objective and subjective continence response as well as quality of life.

## Registration

The registration of this study can be found on PROSPERO International Prospective Register of Systematic Reviews under number CRD42022368652.

## Funding

This research did not receive any specific grant from funding agencies in the public, commercial, or not-for-profit sectors.

## CRediT authorship contribution statement

**Camila Chaves dos Santos Novais:** Conceptualization, Investigation, Data curation, Writing – original draft. **Adélia Regina Oliveira da Rosa Santana:** Writing – review & editing. **Alisson Rodrigo Moura da Paz:** Investigation, Data curation. **Aline Tenório Lins Carnaúba:** Data curation, Writing – review & editing. **Kelly Cristina Lira de Andrade:** Writing – review & editing, Visualization. **Pedro de Lemos Menezes:** Supervision.

## Conflicts of interest

The authors declare no conflicts of interest.

## Appendix Supplementary materials

Image, application 1

Image, application 2
