# Supplementary material for: The impact of using electromyographic biofeedback on pelvic floor rehabilitation in men with post-prostatectomy urinary incontinence: a meta-analysis
Source: Clinics (Sao Paulo). 2025 May 13;80:100687. doi: 10.1016/j.clinsp.2025.100687 (PMC12142337; doi:10.1016/j.clinsp.2025.100687)
Supplement: Supplementary file 1 [file mmc1.docx]

CLINICS-D-24-00579_Supplementary Materials

**Appendix 1** Search algorithm

| **#1** |
| --- |
| (“Urinary Incontinence” [MeSH Terms]) OR “Incontinence problem” |
| AND |
| **#2** |
| (“Prostatectomy [MeSH Terms]” OR “Prostatectomies” OR “Suprapubic Prostatectomies” OR “Suprapubic Prostatectomy” OR “Retropubic Prostatectomies” OR “Retropubic Prostatectomy”) |
| AND |
| **#3** |
| (“Biofeedback, Psychology” [MeSH Terms] OR “Biofeedback” OR “Biofeedbacks” OR “Neurofeedback” [MeSH Terms] OR “Electromyography Feedback” OR “Physical Therapy Modalities” [MeSH Terms] OR “Physical Therapy Modality” OR “Physiotherapy (Techniques)” OR “Physiotherapies (Techniques)” OR “Physical Therapy Techniques” OR “Physical Therapy Technique” OR “Physical Therapy” OR “Physical Therapies” OR “Rehabilitation” [MeSH Terms] OR “Habilitation”) OR “pelvic floor muscle training” |

**Appendix 2** Excluded articles and reason for exclusion (n = 103).

| **Author, Year [Reference]** | **Reason for exclusion^a^** |
| --- | --- |
| Bales et al., 2000 [1] | 1 |
| Bocker and Smolenski, 2002 [2] | 12 |
| Boyd, 1998 [3] | 10 |
| Burgio et al., 2006 [4] | 2 |
| Burgio, Stutzman and Engel 1989 [5] | 2 |
| Centemero et al., 2009 [6] | 10 |
| Centemero et al., 2010 [7] | 3 |
| Ceresoli et al., 2002 [8] | 10 |
| Cherniack, 2006 [9] | 10 |
| Collado Serra et al., 2013 [10] | 10 |
| Cornel, Wit and Witjes, 2005 [11] | 4 |
| Crevenna et al., 2003 [12] | 5 |
| Crivellaro et al., 2011 [13] | 3 |
| de Lira et al., 2019 [14] | 4 |
| de Souza et al., 2022 [15] | 11 |
| Delmastro et al.,2010 [16] | 3 |
| Delneri et al., 2018 [17] | 3 |
| Demidko et al., 2015 [18] | 5 |
| Dijkstra-Eshuis et al., 2015 [19] | 1 |
| Feng et al., 2022 [20] | 8 |
| Fernández-Cuadros et al., 2016 [21] | 5 |
| Fortunato, Schettini and Gallucci, 1996 [22] | 5 |
| García-Sánchez et al., 2022 [23] | 3 |
| Garg et al., 2019 [24] | 5 |
| Gazimiev and Demidko, 2022 [25] | 5 |
| Geanini-Yagüez et al., 2014 [26] | 9 |
| Gezginci, Goktas and Ata et al., 2023 [27] | 3 |
| Glazener et al., 2010 [28] | 10 |
| Glazener et al., 2011 [29] | 8 |
| Glazener et al., 2011 [30] | 3 |
| Glazener et al., 2011 [31] | 10 |
| Glybochko et al., 2013 [32] | 12 |
| Gomes et al., 2018 [33] | 2 |
| Goode et al., 2009 [34] | 10 |
| Goode et al., 2009 [35] | 10 |
| Goode, 2012 [36] | 10 |
| Jackson et al., 1996 [37] | 5 |
| James et al., 2012 [38] | 3 |
| Juszczak et al., 2022 [39] | 10 |
| Kaczmarek, 1995 [40] | 10 |
| Khorrami et al., 2023 [41] | 8 |
| Kim, Lee and Ko, 2021 [42] | 3 |
| Krotova et al., 2021 [43] | 11 |
| Laurienzo et al., 2013 [44] | 3 |
| Laurienzo et al., 2015 [45] | 10 |
| Lazzeri, Guazzoni and Montorsi, 2012 [46] | 10 |
| Lilli et al., 2006 [47] | 1 |
| Loi, 2014 [48] | 10 |
| Majima et al., 2018 [49] | 10 |
| Mallol-Badellino et al., 2015 [50] | 5 |
| Marchiori et al., 2010 [51] | 3 |
| Martini et al., 2011 [52] | 3 |
| Mathewson-Chapman, 1995 [53] | 10 |
| Mathewson-Chapman, 1997 [54] | 7 |
| Milios, Ackland and Green, 2019 [55] | 3 |
| Milios, Ackland and Green, 2018 [56] | 10 |
| Milonas et al., 2018 [57] | 10 |
| Moore, Griffiths and Hughton, 1999 [58] | 3 |
| Moriconi et al., 2022 [59] | 10 |
| Morihiro et al., 2011 [60] | 10 |
| Morkved et al., 2008 [61] | 10 |
| Overgard et al., 2008 [62] | 2 |
| Parekh et al., 2003 [63] | 4 |
| Pedriali et al., 2014 [64] | 10 |
| Pedriali et al., 2016 [65] | 2 |
| Perez et al., 2018 [66] | 2 |
| Prota et al., 2012 [67] | 14 |
| Prota et al., 2009 [68] | 10 |
| Rajkowska-Labon, Skrobot and Bakula, 2006 [69] | 5 |
| Ribeiro et al., 2008 [70] | 10 |
| Ribeiro et al., 2009 [71] | 10 |
| Robinson et al., 2008 [72] | 3 |
| Robinson et al., 2009 [73] | 10 |
| Sacco et al., 2011 [74] | 8 |
| Sacco et al., 2012 [75] | 10 |
| Salciccia et al., 2021 [76] | 5 |
| Sangalli et al.,2021 [77] | 10 |
| Sayılan and Özbas, 2018 [78] | 3 |
| Sayner et al., 2018 [79] | 10 |
| Sayner, 2019 [80] | 3 |
| Sciarra et al., 2009 [81] | 10 |
| Serdà and Marcos-Gragera, 2014 [82] | 3 |
| Strojek et al., 2021 [83] | 3 |
| Sujka, Kaczmarek and Ricker, 1998 [84] | 10 |
| Tafuri et al., 2018 [85] | 10 |
| Tienforti et al., 2012 [86] | 3 |
| Van Kapen et al., 2000 [87] | 4 |
| Veshnavei, 2021 [88] | 8 |
| Vinarov et al., 2018 [89] | 5 |
| Voorham-van Der Zalmet et al., 2013 [90] | 10 |
| Wang et al., 2018 [91] | 10 |
| Wille et al., 2003 [92] | 4 |
| Xiao et al., 2008 [93] | 5 |
| Yamanishi et al., 2007 [94] | 3 |
| Yang et al., 2010 [95] | 3 |
| Yang et al., 2020 [96] | 4 |
| Zellner, 2012 [97] | 12 |
| Zerman et al., 2000 [98] | 10 |
| Zhang et al. 2013 [99] | 10 |
| Zhang et al., 2006 [100] | 8 |
| Zhang et al., 2015 [101] | 13 |
| Zhang, 2013 [102] | 10 |
| Zhang, Strauss and Siminoff, 2007 [103] | 8 |

^a^1. EMG-BFB preoperative only; 2. Pressure EMG-BFB; 3. Does not use EMG-BFB; 4. Undefined BFB type; 5. Non-randomized clinical trial; 6. Does not compare with EMG-BFB; 7. EMG-BFB only in the evaluation; 8. Use of EMG-BFB on an occasional basis; 9. Sample with men and women; 10. Abstract file; 11. Review; 12. Article not found and no response from the authors; 13. Condition other than prostatectomy; 14. Therapy applied to conditions other than UI.

Source: Prepared by the authors.

References

1. Bales GT, Gerber GS, Minor TX, Mhoon DA, McFarland JM, Kim HL, et al. Effect of preoperative biofeedback/pelvic floor training on continence in men undergoing radical prostatectomy. Urology. 2000;56(4):627-30.

2. Bocker B, Smolenski UC. Physikalische therapie der beckenbodeninsuffizienz: methodenvergleich [Physical therapy of pelvic floor insufficiency: comparison of methods]. J Urol Urogynakol. 2002;(2):20-7. German.

3. Boyd LA. The effects of biofeedback on the occurrence of urinary incontinence in patients following radical retropubic prostatectomy [thesis]. Florida: Florida International University; 1998.

4. Burgio KL, Goode PS, Urban DA, Umlauf MG, Locher JL, Bueschen A, et al. Preoperative biofeedback assisted behavioral training to decrease post-prostatectomy incontinence: a randomized, controlled trial. J Urol. 2006;175(1):196-201.

5. Burgio KL, Stutzman RE, Engel BT. Behavioral training for post-prostatectomy urinary incontinence. J Urol. 1989;141(2):303-6.

6. Centemero A, Rigatti L, Andrea L, Gallina A, Lughezzani G, Montorsi F, et al. Effectiveness of pre-operative pelvic floor muscle training for post-prostatectomy early continence recovery. Journal of Urology. 2009;181(4S):591.

7. Centemero A, Rigatti L, Giraudo D, Lazzeri M, Lughezzani G, Zugna D, et al. Preoperative pelvic floor muscle exercise for early continence after radical prostatectomy: a randomised controlled study. Eur Urol. 2010;57(6):1039-44.

8. Ceresoli A, Goumas JK, Colombo F, Barbetti E, Dell’Aglio F, Bonacina P, et al. Daily transcutaneous electrical nerve stimulation (DTENS) after radical perineal prostatectomy: a free cost-effective biofeedback technique in the treatment of post operative urinary incontinence. Poster session presented at: International Continence Society [Internet]. 2002 Aug 28-30; Heidelberg. Available from: https://www.ics.org/2002/abstract/426.

9. Cherniack EP. Biofeedback and other therapies for the treatment of urinary incontinence in the elderly. Altern Med Rev. 2006;11(3):224-31.

10. Collado Serra A, Cabo MP, Backhaus MR, Dominguez-Escrig J, Rubio-Briones J, Gomez-Ferrer A, et al. Intensive preoperatory Pelvic Floor Muscle Training reduce duration and severity of stress urinary incontinence after radical prostatectomy: a randomized controlled trial. Eur Urol Suppl. 2013;12(1):e1007-e1008.

11. Cornel EB, de Wit R, Witjes JA. Evaluation of early pelvic floor physiotherapy on the duration and degree of urinary incontinence after radical retropubic prostatectomy in a non-teaching hospital. World J Urol. 2005;23(5):353-5.

12. Crevenna R, Zoch C, Keilani M, Quittan M, Fialka-Moser V. Implementation of a physical rehabilitation group for post-prostatectomy urinary incontinence patients and its effects on quality of life. Phys Med Rehab Kuror. 2003;13(6):339-44.

13. Crivellaro S, Abbinante M, Martinez G, Tosco L, Palazzetti A, Frea B. Efficacy of ultrasound-guided pelvic muscle training. Neurourol Urodyn. 2011;30(6):1013-4.

14. de Lira GHS, Fornari A, Cardoso LF, Aranchipe M, Kretiska C, Rhoden EL. Effects of perioperative pelvic floor muscle training on early recovery of urinary continence and erectile function in men undergoing radical prostatectomy: a randomized clinical trial. Int Braz J Urol. 2019;45(6):1196-1203.

15. de Souza JPM, Silva MCS, Oliveira VLS, da Silva RA, de Oliveira GGB, Soares BOS, et al. Importância do treinamento da musculatura do assoalho pélvico associado ao biofeedback em pacientes com incontinência urinária após prostatectomia [Importance of pelvic floor muscle training associated with biofeedback in patients with urinary incontinence after prostatectomy]. Braz J Health Rev. 2022;5(5):19774-9. Portuguese.

16. Delmastro F, Marchisio C, Gianfranco L, Donatella G. Urinary incontinence after radical prostatectomy: A randomized controlled trial comparing preoperative intensive pelvic muscle exercises with or without proprioceptive training. Neurourol Urodyn. 2010;29(S2):62-3.

17. Delneri C, Iona L, Giorgini T, Zampa A, Tasso L, Cattarossi L, et al. Urinary incontinence after radical prostatectomy: When the rehabilitation treatment? Neurourol Urodyn. 2018; 37(S3):66-67.

18. Demidko YL, Glybochko PV, Vinarov AZ, Rapoport LM, Chaly ME, Akhvlediani ND, et al. [Treatment of urinary incontinence after radical prostatectomy using training of pelvic muscles under the control of biofeedback]. Urologiia. 2015;(1):41-3. Russian.

19. Dijkstra-Eshuis J, Van den Bos TW, Splinter R, Bevers RFM, Zonneveld WCG, Putter H, et al. Effect of preoperative pelvic floor muscle therapy with biofeedback versus standard care on stress urinary incontinence and quality of life in men undergoing laparoscopic radical prostatectomy: a randomised control trial. Neurourol Urodyn. 2015;34(2):144-50.

20. Feng X, Lv J, Li M, Lv T, Wang S. Short-term efficacy and mechanism of electrical pudendal nerve stimulation versus pelvic floor muscle training plus transanal electrical stimulation in treating post-radical prostatectomy urinary incontinence. Urology. 2022;160:168-75.

21. Fernández-Cuadros ME, Nieto-Blasco J, Geanini-Yagüez A, Ciprián-Nieto D, Padilla-Fernández B, Lorenzo-Gómez MF. Male urinary incontinence: associated risk factors and electromyography biofeedback results in quality of life. Am J Mens Health. 2016;10(6):NP127-35.

22. Fortunato P, Schettini M, Gallucci M. The rehabilitation of the perineal floor by biofeedback and functional electrostimulation in the treatment of urinary incontinence following radical prostatectomy. Preliminary results. Acta Urologica Italica. 1996;10(4):281-4.

23. García-Sánchez C, García-Obrero I, Barrero-Candau R, García-Ramos JB, Rodríguez-Pérez AJ, Medina-López RA. Randomized and open trial to assess the effectiveness of the guided pelvic floor exercises pre-radical robotic prostatectomy on the improvement of urinary incontinence. Initial results. Arch Esp Urol. 2022;75(6):544-51.

24. Garg V, Sudarshan SB, Ganpule A, Mohankumar V, Singh A, Sabnis RB, et al. Continence after radical prostatectomy: it's not just nerve preservation. Indian J Urol. 2019;35(S1):S35.

25. Gazimiev AM, Demidko YL. [Rehabilitation of urinary continence in patients 5 after laparoscopic radical prostatectomy]. Urologiia. 2022(3):15-8. Russian.

26. Geanini-Yagüez A, Fernández-Cuadros ME, Nieto-Blasco J, Ciprián-Nieto D, Oliveros-Escudero B, Lorenzo-Gómez MF. EMG-biofeedback en el tratamiento de la incontinencia urinaria y calidad de vida [Electromiography-biofeedback in the treatment of urinary incontinence and quality of life]. Rehabilitacion. 2014;48(1):17-24. Spanish.

27. Gezginci E, Goktas S, Ata A. Effect of perioperative pelvic floor muscle training program on incontinence and quality of life after radical prostatectomy: a randomized controlled trial. Clin Rehabil. 2023;37(4):534-44.

28. Glazener C, Boachie C, Buckley B, Cochran C, Dorey G, Grant A, et al. A randomised controlled trial of conservative treatment (pelvic floor muscle training and bladder training) for urinary incontinence in men after prostate surgery (maps). Neurourol Urodyn. 2010;29(6):1093-4.

29. Glazener C, Boachie C, Buckley B, Cochran C, Dorey G, Grant A, et al. Conservative treatment for urinary incontinence in Men After Prostate Surgery (MAPS): two parallel randomised controlled trials. Health Technol Assess. 2011;15(24):1-290, iii-iv.

30. Glazener C, Boachie C, Buckley B, Cochran C, Dorey G, Grant A, et al. Urinary incontinence in men after formal one-to-one pelvic-floor muscle training following radical prostatectomy or transurethral resection of the prostate (MAPS): two parallel randomised controlled trials. Lancet. 2011;378(9788):328-37.

31. Glazener C, Boachie C, Hagen S, Kilonzo M, Cochran C, Buckley, et al. Clinical outcomes two years after a randomised controlled trial of pelvic floor muscle training after radical prostatectomy or turp: Men After Prostate Surgery trial (MAPS). Neurourol Urodyn. 2011;30(6):1150-1.

32. Glybochko P, Alyaev Y, Vinarov A, Chalyi M, Demidko Y, Myannik S, et al. [Biofeedback pelvic muscle training in patients with urinary incontinence following radical prostatectomy]. Vrach [Internet]. 2013;24(1):61-5. Russian.

33. Gomes CS, Pedriali FR, Urbano MR, Moreira EH, Averbeck MA, Almeida SHM. The effects of Pilates method on pelvic floor muscle strength in patients with post-prostatectomy urinary incontinence: a randomized clinical trial. Neurourol Urodyn. 2018;37(1):346-53.

34. Goode P, Burgio K, Johnson T, Roth D, Clay O, Burkhardt J, et al. Behavioral therapy with or without biofeedback and pelvic floor electrical stimulation for persistent post-prostatectomy incontinence: a randomized controlled trial. Neurourol Urodyn. 2009;28(7):681-2.

35. Goode PS, Burgio KL, Johnson TM, Roth DL, Clay OJ, Burkhardt JH, et al. Pelvic floor electrical stimulation, biofeedback, and behavioral therapy for persistent post-prostatectomy incontinence. J Urol. 2009;181(4):591-2.

36. Goode PS. Efficacy of an assisted low-intensity programme of perioperative pelvic floor muscle training in improving the recovery of continence after radical prostatectomy: a randomized controlled trial. BJU Int. 2012;110(7):1010-1.

37. Jackson J, Emerson L, Johnston B, Wilson J, Morales A. Biofeedback: a noninvasive treatment for incontinence after radical prostatectomy. Urol Nurs. 1996;16(2):50-4.

38. James MH, Gibbs BB, Glace E, Given RW. Influence of preoperative pelvic floor muscle strength on post-prostatectomy incontinence. J Urol. 2012;187(4S):e545.

39. Juszczak A, Konecki T, Kutwin P, Roman L, Cichocki M, Jabłonowski Z. Prerehabilitation can improve urinary continence in patients after laparoscopic radical prostatectomy. J Urol. 2022;207:e497-8.

40. Kaczmarek P. Biofeedback in the treatment of urinary-incontinence post radical prostatectomy. Biofeedback and self-regulation. 1995;20(3):289-90.

41. Khorrami MH, Mohseni A, Gholipour F, Alizadeh F, Zargham M, Izadpanahi MH, et al. Single session pre-operative pelvic floor muscle training with biofeedback on urinary incontinence and quality of life after radical prostatectomy: a randomized controlled trial. Urol Sci. 2023;34(1):23-7.

42. Kim YU, Lee DG, Ko YH. Pelvic floor muscle exercise with biofeedback helps regain urinary continence after robot-assisted radical prostatectomy. Yeungnam Univ J Med. 2021;38(1):39-46.

43. Krotova NO, Ulitko TV, Kuzmin IV, Al-Shukri SK. Biofeedback in the treatment of patients with urine incontinence after radical prostatectomy. Urology reports (St. Petersburg). 2021;11(1):69-78.

44. Laurienzo CE, Sacomani CAR, Rodrigues TR, Zequi SC, Guimarães GC, Lopes A. Results of preoperative electrical stimulation of pelvic floor muscles in the continence status following radical retropubic prostatectomy. Int Braz J Urol. 2013;39(2):182-8. d

45. Laurienzo C, Magnabosco W; Jabur F, Gameiro M, Yamamoto H, Guerra R, et al. Post-prostatectomy urinary incontinence and erectile dysfunction: The role of pelvic floor rehabilitation. Neurourol Urodyn. 2015;34:S449-50.

46. Lazzeri M, Guazzoni G, Montorsi F. Pelvic floor muscle training after prostate surgery. Lancet. 2012;379(9811):120-1.

47. Lilli P, Mercuriali M, Fiori M, Hanitzsch H, Gunelli R, Bercovich E. Impact of preoperative biofeedback on incontinence in cancer patients undergoing radical prostatectomy. Arch Ital Urol Androl. 2006;78(3):92-6.

48. Loi NGS. A randomised controlled trial study of the efficacy of intensive pre-operative pelvic floor muscle training to decrease post-prostatectomy urinary incontinence. Int J Urol. 2014;21(S2):A169.

49. Majima K, Nagasawa R, Seki M, Kouchi Y, Muromiya Y, Omoto K, et al. Possible impact of continuous physiotherapist guided pelvic floor muscle training to reduce urinary incontinence after robot-assisted laparo-scopic prostatectomy. Int J Urol. 2018;25(S1):404.

50. Mallol-Badellino J, Sánchez-Fabero A, Mateo-Lozano S, Martín-Baranera M, Moreno-Atanasio E, Tinoco-González J. Resultados en la calidad de vida y la severidad de la incontinencia urinaria tras rehabilitación en varones prostatectomizados por neoplasia de próstata [Results of rehabilitation on quality of life and urinary incontinence severity after radical prostatectomy]. Rehabilitacion. 2015;49(4):210-5. Spanish.

51. Marchiori D, Bertaccini A, Manferrari F, Ferri C, Martorana G. Pelvic floor rehabilitation for continence recovery after radical prostatectomy: role of a personal training re-educational program. Anticancer Res [Internet]. 2010;30(2):553-6.

52. Martini M, Bernardini S, Blanc E, Piretta K, Tappero R. Relationship between integrity of pelvic floor function and recovery of continence after laparoscopic prostatectomy and effects of preventive pelvic floor training inmaleswith pelvic floor weakness. Neurourol Urodyn. 2011;30(S1):11-2.

53. Mathewson-Chapman M. The effect of pelvic muscle exercises with biofeedback for urinary incontinence post-prostatectomy. University of Florida. 1995.

54. Mathewson-Chapman M. Pelvic muscle exercise/biofeedback for urinary incontinence after prostatectomy: an education program. J Cancer Educ. 1997;12(4): 218-23.

55. Milios JE, Ackland TR, Green DJ. Pelvic floor muscle training in radical prostatectomy: a randomized controlled trial of the impacts on pelvic floor muscle function and urinary incontinence. BMC Urol. 2019;19(1):116.

56. Milios JE, Ackland TR, Green DJ. New protocols for a faster return to continence and quality of life following radical prostatectomy. BJU Int. 2018;122(S2):17.

57. Milonas D, Siupsinskas L, Zachovajevas P, Zachovajieviene B. Effectiveness of different postoperative training programs on pelvic floor muscles strengthening and reducing of urinary incontinence in men after radical prostatectomy: results of randomized controlled clinical trial. Eur Urol Suppl. 2018;17(5):e2199.

58. Moore KN, Griffiths D, Hughton A. Urinary incontinence after radical prostatectomy: a randomized controlled trial comparing pelvic muscle exercises with or without electrical stimulation. BJU Int. 1999;83(1):57-65.

59. Moriconi M, Salciccia S, Del Giudice F, Viscuso P, Canale V, Rosati D, et al. How to predict outcomes from a biofeedback and pelvic floor muscle electric stimulation program in patients with urinary incontinence after radical prostatectomy. EU Open Science. 2022;44:S114.

60. Morihiro N, Masatsugu I, Shinji K, Kenichi T, Kazumasa M, Shiro B. Effectiveness of Sacral Surface Therapeutic Electrical Stimulation (SSTES) on early recovery of urinary incontinence after laparoscopic radical prostatectomy: a prospective study. Neurourol Urodyn. 2011;30(6):889-90.

61. Morkved S, Overgard M, Lydersen S, Angelsen A. Does pelvic floor muscle training with follow up instructions by a physiotherapist reduce urinary incontinence after radical prostatectomy? A randomised controlled trial. Neurourol Urodyn. 2008;27(7):587-8.

62. Overgard M, Angelsen A, Lydersen S, Morkved S. Does physiotherapist-guided pelvic floor muscle training reduce urinary incontinence after radical prostatectomy? A randomised controlled trial. Eur Urol. 2008;54(2):438-48.

63. Parekh AR, Feng MI, Kirages D, Bremner H, Kaswick J, Aboseif S. The role of pelvic floor exercises on post-prostatectomy incontinence. J Urol. 2003;170(1):130-3.

64. Pedriali F, Gomes C, Soares L, Urbano M, Moreira E, de Almeida S. The efficacy of pilates compared to pelvic floor muscle training associated with electrical stimulation in the recovery of post-prostatectomy urinary incontinence: a randomized controlled trial. Neurourol Urodyn. 2014;33(6):742-3.

65. Pedriali FR, Gomes CS, Soares L, Urbano MR, Moreira EC, Averbeck MA, et al. Is pilates as effective as conventional pelvic floor muscle exercises in the conservative treatment of post-prostatectomy urinary incontinence? A randomised controlled trial. Neurourol Urodyn. 2016;35(5):615-21.

66. Perez FSB, Rosa NC, da Rocha AF, Peixoto LRT, Miosso CJ. Effects of biofeedback in preventing urinary incontinence and erectile dysfunction after radical prostatectomy. Front Oncol. 2018;8:20.

67. Prota C, Gomes CM, Ribeiro LH, de Bessa J Jr, Nakano E, Dall'Oglio M, et al. Early postoperative pelvic-floor biofeedback improves erectile function in men undergoing radical prostatectomy: a prospective, randomized, controlled trial. Int J Impot Res. 2012;24(5):174-8.

68. Prota C, Ribeiro LS, Gomes CM, Bessa J, Boldarine MP, Nakano E, et al. Early pelvic-floor biofeedback training promotes improvement of erectile function after radical prostatectomy. J Urol. 2009;181(4S):524-5.

69. Rajkowska-Labon E, Skrobot W, Bakuła S. [Evaluating the effect of conservative physiotherapeutical treatment on urine continence in males after radical prostatectomy: a preliminary report]. Family Medicine and Primary Care Review. 2006;8(4):1288-93. Polish.

70. Ribeiro LS, Prota C, Gomes CM, Dall'Oglio MF, Bruschini H, Srougi M. Effect of early postoperative pelvic-floor biofeedback on continence in men undergoing radical prostatectomy: a randomized, controlled trial. J Urol. 2008;179(4):483.

71. Ribeiro LS, Prota C, Gomes CM, Boldarine MP, Nakano E, Dall'Oglio M, et al. Early pelvic-floor biofeedback training promotes long-term improvement of urinary continence after radical prostatectomy. J Urol. 2009;181(4S):680.

72. Robinson JP, Bradway CW, Nuamah I, Pickett M, McCorkle R. Systematic pelvic floor training for lower urinary tract symptoms post-prostatectomy: a randomized clinical trial. Int J Urol Nurs. 2008;2(1):3-13.

73. Robinson J, Weiss R, Avi-Itzhak T, McCorkle R. Pilot-testing of a theory-based pelvic floor training intervention for radical prostatectomy patients. Neurourol Urodyn. 2009;28(7):682-3.

74. Sacco E, Tienforti D, D'Addessi A, Racioppi M, Gulino G, Pinto F, et al. Efficacy of a supervised, affordable program of perioperative pelvic floor muscle training in improving the recovery of continence after radical prostatectomy: a randomized controlled trial. Neurourol Urodyn. 2011;30(6):995-7.

75. Sacco E, Tienforti D, Marangi F, D'Addessi A, Racioppi M, Gulino G, et al. Efficacy of a supervised low-intensity regimen of perioperative pelvic floor muscle training in reducing postprostatectomy urinary incontinence: a randomized controlled trial. Eur Urol Suppl. 2012;11(1):e286.

76. Salciccia S, Sciarra A, Moriconi M, Maggi M, Viscuso P, Rosati D, et al. How to predict outcomes from a biofeedback and pelvic floor muscle electric stimulation program in patients with urinary incontinence after radical prostatectomy. J Clin Med. 2021;11(1):127.

77. Sangalli MN, Vota P, Zanoni M, Toia G, Mazzieri C, Mandressi A, et al. Randomized trial comparingurinary continence rates between pelvic muscles exercises with and without trans-pelvic magnetic stimulation after robotic assisted radical prostatectomy. J Urol. 2021;206(3S):e1068.

78. Sayılan AA, Özbaş A. The effect of pelvic floor muscle training on incontinence problems after radical prostatectomy. Am J Mens Health. 2018;12(4):1007-15.

79. Sayner A, Nahon I, Davies S, Haines K, Karahalios E, Ogluszko C. Pre-operative functional pelvic floor muscle training in radical prostatectomy: identifying feasibility. BJU Int. 2018;122:20-1.

80. Sayner A. Functional pelvic floor muscle training before radical prostatectomy: a prospective randomised controlled pilot study. Neurourol Urodyn. 2019;38:S348-50.

81. Sciarra A, Salciccia S, Gentilucci A, Alfarone A, Di Pierro GB, Mariotti G, et al. Early recovery of urinary continence after radical prostatectomy using early pelvic floor electric stimulation and biofeedback associated treatment. J Urol. 2009;181(4):680.

82. Serdà BCF, Marcos-Gragera R. Urinary incontinence and prostate cancer: a progressive rehabilitation program design. Rehabil Nurs. 2014;39(6):271-80.

83. Strojek K, Weber-Rajek M, Straczynska A, Piekorz Z, Pilarska B, Jarzemski P, et al. Randomized-controlled trial examining the effect of pelvic floor muscle training in the treatment of stress urinary incontinence in men after a laparoscopic radical prostatectomy pilot study. J Clin Med. 2021;10(13):2946.

84. Sujka SK, Kaczmarek P, Ricker L. Efficacy of EMG biofeedback treatment for post-radical prostatectomy urinary incontinence. *In:* 29th Annual Meeting of the Association for Applied Psychophysiology and Biofeedback; 1998, April 1-5; Florida. p. 132.

85. Tafuri A, Bassi S, Sebben M, Pirozzi M, Balzarro M, Porcaro AB, et al. A pilot randomized trial of preoperative pelvic floor muscle exercise vs usual care to improve sexual function and health related quality of live after RARP: preliminary disappointed results. Neurourol Urodyn. 2018;37(S3):S7-8.

86. Tienforti D, Sacco E, Marangi F, D'Addessi A, Racioppi M, Gulino G, et al. Efficacy of an assisted low-intensity programme of perioperative pelvic floor muscle training in improving the recovery of continence after radical prostatectomy: a randomized controlled trial. BJU Int. 2012;110(7):1004-10.

87. Van Kampen M, De Weerdt W, Van Poppel H, De Ridder D, Feys H, Baert L. Effect of pelvic-floor re-education on duration and degree of incontinence after radical prostatectomy: a randomised controlled trial. Lancet. 2000;355(9198):98-102.

88. Veshnavei HA. Urinary incontinency after radical prostatectomy and effects of 1-month pre-operative biofeedback training. Am J Clin Exp Urol. 2021;9(6):489-96.

89. Vinarov AZ, Rapoport LM, Krupinov GE, Demidko YL, Tsarichenko DG, Bezrukov EA, et al. [Biofeedback-assisted pelvic floor muscle training in patients with urinary incontinence after laparoscopic and robot-assisted radical prostatectomy]. Onkourologiya. 2018;14(2):102-8. Russian.

90. Voorham-van der Zalm P, Dijkstra-Eshuis J, Splinter R, Putter H, Bevers RFM, Pelger RCM. Effect of preoperative pelvic floor muscle therapy versus standard care on stress urinary incontinence in men undergoing radical laparoscopic prostatectomy. J Urol. 2013;189(4S):e558-9.

91. Wang S, Lv J, Li M, Lv T. Efficacy and mechanism of electrical pudendal nerve stimulation in treating post-radical prostatectomy urinary incontinence. Neurourol Urodyn. 2018;37:S324-5.

92. Wille S, Sobottka A, Heidenreich A, Hofmann R. Pelvic floor exercises, electrical stimulation and biofeedback after radical prostatectomy: results of a prospective randomized trial. J Urol. 2003;170(2):490-3.

93. Xiao HJ, Gao X, Cai YB, Zhou XF, Qiu JG, Wen XQ, et al. Biofeedback electrical stimulation for treatment of urinary incontinence after laparoscopic radical prostatectomy. J Clin Rehabilitative Tissue Engineering Res. 2008;12(9):1722-4.

94. Yamanishi T, Mizuno T, Watanabe M, Honda M, Yoshida KI. Randomized, placebo controlled study of electrical stimulation with pelvic floor muscle training for severe urinary incontinence after radical prostatectomy. J Urol. 2010;184(5):2007-12.

95. Yang BS, Ye DW, Yao XD, Peng JY, Zhang SL, Dai B, et al. [The study of electrical acupuncture stimulation therapy combined with pelvic floor muscle therapy for postprostatectomy incontinence]. Zhonghua Wai Ke Za Zhi. 2010;48(17):1325-7. Chinese.

96. Yang H, Ting-Ting L, Xiao L, Yao LJ, Jian-Wei L. [Electroacupuncture stimulation of the pudendal nerve for urinary incontinence after radical prostatectomy]. Zhonghua Nan Ke Xue. 2020;26(12):1119-23. Chinese.

97. Zellner M. [Comparison of efficiency of physical therapy, combined electro stimulation and biofeedback device, and whole-body vibration therapy for the treatment of incontinence after radical prostatectomy]. J Urol Urogynakol. 2012;19(2):8-15. German.

98. Zermann DH, Wunderlich H, Reichelt O, Schubert J. Re: early post-prostatectomy pelvic floor biofeedback. J Urol. 2000;164(3):783-4.

99. Zhang A, Bodner D, Fu A, Gordon N, Klein E, Kresevic D, et al. A patient-centered approach to persistent urinary incontinence in prostate cancer patients. Psycho-Oncology. 2013;22(S3):66.

100. Zhang AY, Strauss GJ, Siminoff LA. Intervention of urinary incontinence and quality of life outcome in prostate cancer patients. J Psychosoc Oncol. 2006;24(2):17-30.

101. Zhang AY, Bodner DR, Fu AZ, Gunzler DD, Klein E, Kresevic D, et al. Effects of patient centered interventions on persistent urinary incontinence after prostate cancer treatment: a randomized, controlled trial. J Urol. 2015;194(6):1675-81.

102. Zhang A. The problem-solving therapy and urinary incontinence in prostate cancer patients. Psycho-Oncology. 2013;22(S2):33-4.

103. Zhang AY, Strauss GJ, Siminoff LA. Effects of combined pelvic floor muscle exercise and a support group on urinary incontinence and quality of life of postprostatectomy patients. Oncol Nurs Forum. 2007;34(1):47-53.

**Appendix 3** JBI critical appraisal tool (JBI) developed by joanna briggs institute for randomized clinical trials.

JBI Critical Appraisal Checklist for randomized controlled trials

Reviewer ______________________________Date__ _____________________

Author____________________________Year_________ Record Number_________

|  | Yes | At the | Unclear | AT |
| --- | --- | --- | --- | --- |
| 1. Was true randomization used for assigning participants to treatment groups? | □ | □ | □ | □ |
| 1. Was allocation to treatment groups concealed? | □ | □ | □ | □ |
| 1. Were treatment groups similar at the baseline? | □ | □ | □ | □ |
| 1. Were participants blind to treatment assignment? | □ | □ | □ | □ |
| 1. Were those delivering treatment blind to treatment assignment? | □ | □ | □ | □ |
| 1. Were outcomes assessors blind to treatment assignment? | □ | □ | □ | □ |
| 1. Were treatment groups treated identically other than the intervention of interest? | □ | □ | □ | □ |
| 1. Was follow up complete and if not, were differences between groups in terms of their follow up adequately described and analyzed? | □ | □ | □ | □ |
| 1. Were participants analyzed in the groups to which they were randomized? | □ | □ | □ | □ |
| 1. Were outcomes measured in the same way for treatment groups? | □ | □ | □ | □ |
| 1. Were outcomes measured in a reliable way? | □ | □ | □ | □ |
| 1. Was appropriate statistical analysis used? | □ | □ | □ | □ |
| 1. Was the trial design appropriate, and any deviations from the standard RCT design (individual randomization, parallel groups) accounted for in the conduct and analysis of the trial? | □ | □ | □ | □ |

Overall appraisal: Include □ Exclude □ Seek further info □

Comments (Including reason for exclusion)

_____________________________________________________________________________________
