# Supplementary material for: The impact of using electromyographic biofeedback on pelvic floor rehabilitation in men with post-prostatectomy urinary incontinence: a meta-analysis
Source: Clinics (Sao Paulo). 2025 May 13;80:100687. doi: 10.1016/j.clinsp.2025.100687 (PMC12142337; doi:10.1016/j.clinsp.2025.100687)
Supplement: Supplementary file 2 [file mmc2.docx]

**The impact of using electromyographic biofeedback on pelvic floor rehabilitation in men with post-prostatectomy urinary incontinence: a meta-analysis**

Camila Chaves dos Santos Novais^a^

**Corresponding author**

Full postal address: Alagoas State University of Health Sciences. Av. Jorge Lima, 113 – Trapiche, CEP: 57010-300, Maceió, Alagoas, Brazil.

E-mail: mila_v2@hotmail.com

OrcID: https://orcid.org/0000-0002-3317-9719

Adélia Regina Oliveira da Rosa Santana^a^

E-mail: adeliarosa.fisio@gmail.com

OrcID: <https://orcid.org/0000-0002-4221-2218>

Alisson Rodrigo Moura da Paz^b^

E-mail: alissonrodriigo.fisio@outlook.com

OrcID: <https://orcid.org/0009-0005-0402-3984>

Aline Tenório Lins Carnaúba^a^

E-mail: aline.carnauba@uncisal.edu.br

OrcID: <https://orcid.org/0000-0003-4100-6866>

Kelly Cristina Lira de Andrade^a^

E-mail: kellyclandrade@gmail.com

OrcID: <https://orcid.org/0000-0002-2618-4958>

Pedro de Lemos Menezes^a,c^

E-mail: [pedrodelemosmenezes@gmail.com](mailto:pedrodelemosmenezes@gmail.com)

OrcID: <https://orcid.org/0000-0003-1999-5055>

^a^ Alagoas State University of Health Sciences (UNCISAL). Av. Jorge Lima, 113 – Trapiche, CEP: 57010-300, Maceió, Alagoas, Brasil.

^b^ Faculdade Estácio de Alagoas. Av. Pio XII, 70 - Jatiúca, CEP: 57035-560, Maceió, Alagoas, Brasil.

^c^ Federal University of Alagoas (UFAL), Northeast Biotechnology Network (RENORBIO). Av. Lourival Melo Mota, S/N, Tabuleiro do Martins, CEP: 57072-900, Maceió, Alagoas, Brasil.
